# Long-Term Metabolic Remission and Predictive Factors After Sleeve Gastrectomy and Roux-en-Y Gastric Bypass in an Asian Population

**DOI:** 10.3390/jcm15041539

**Published:** 2026-02-15

**Authors:** Kanittha Sakolprakaikit, Kamthorn Yolsuriyanwong, Siripong Cheewatanakornkul, Piyanun Wangkulangkul, Rattana Leelawattana, Pirun Saelue, Darawan Promchan, Praisuda Bualoy

**Affiliations:** 1 Songklanagarind Excellence Center for Obesity and Metabolic Surgery, Division of Surgery, Songklanagarind Hospital, Faculty of Medicine, Prince of Songkla University, Songkhla 90110, Thailand; fmispsu@gmail.com (K.S.); siripong.c@psu.ac.th (S.C.); piyanun.w@psu.ac.th (P.W.); 2 Division of Internal Medicine, Songklanagarind Hospital, Faculty of Medicine, Prince of Songkla University, Songkhla 90110, Thailand; rattanaleelawattana@gmail.com (R.L.); pirun2118@hotmail.com (P.S.); 3 Department of Surgical Nursing, Faculty of Nursing, Prince of Songkla University, Songkhla 90110, Thailand; pdarawann@hotmail.com (D.P.); bprisuda@medicine.psu.ac.th (P.B.)

**Keywords:** bariatric surgery, long-term, metabolic remission, predictive factors, Asian population, sleeve gastrectomy, Roux-en-Y gastric bypass, observational study

## Abstract

**Background/Objective:** Bariatric surgery is an established treatment for individuals with severe obesity, providing sustained weight loss and improvement in obesity-related comorbidities. However, evidence on long-term outcomes and predictors of metabolic resolution, particularly among Asian populations, remains limited. We aimed to evaluate metabolic outcomes after bariatric surgery and identify predictive factors associated with remission. **Methods:** We retrospectively reviewed the data of 581 patients who underwent laparoscopic sleeve gastrectomy (SG) or Roux-en-Y gastric bypass (RYGB) at a tertiary care center between January 2012 and December 2022. Surgical techniques, postoperative follow-up, and baseline characteristics were recorded. Remission and improvement of metabolic comorbidities were assessed during 1–5 years of follow-up. Predictive factors were analyzed, and remission rates between SG and RYGB were compared using propensity score matching. **Results:** A total of 154 (26.5%) individuals had type 2 diabetes mellitus (T2DM), 162 (27.8%) hypertension (HT), 173 (29.7%) dyslipidemia (DLP), and 407 (70.0%) metabolic syndrome (MetS). Remission occurred in 79.1% of individuals with T2DM, 36.0% with HT, 33.9% with DLP, and 79.6% with MetS. Predictive factors included T2DM duration < 3 years, younger age for HT and DLP remission, male sex, body mass index < 43 kg/m^2^, and fasting blood glucose level < 126 mg/dL for MetS. RYGB achieved higher remission of DLP than did SG, whereas other outcomes were comparable. **Conclusions:** Bariatric surgery effectively improves metabolic comorbidities, and several predictive factors influence outcomes. RYGB resulted in superior remission of DLP, while other metabolic outcomes were comparable between the two procedures.

## 1. Introduction

Obesity has become a global epidemic, exerting substantial effects on individual health and healthcare systems worldwide [1]. Bariatric surgery is an effective treatment option for individuals with severe obesity, providing long-term weight loss and improvement in obesity-related comorbidities [2,3,4]. Metabolic syndrome (MetS), characterized by insulin resistance, abnormal cholesterol levels, elevated blood pressure, elevated glucose levels, and obesity, poses a considerable global health risk, increasing the risks of cardiovascular disease (CVD) three-fold, type 2 diabetes mellitus (T2DM) five to seven-fold, and all-cause mortality by 1.5-fold [5]. Bariatric procedures such as Roux-en-Y gastric bypass (RYGB) and sleeve gastrectomy (SG) modify the gastrointestinal anatomy and physiology, resulting in alterations in hormonal regulation, nutrient absorption, and metabolic pathways. Previous studies have demonstrated favorable outcomes following bariatric surgery, including substantial weight loss and resolution of metabolic abnormalities such as T2DM (20–90%), hypertension (HT) (45–80%), and dyslipidemia (DLP) (30–80%) [2,6].

Information on long-term weight loss and metabolic outcomes after bariatric surgery in Asian populations remains limited, and the metabolic changes and predictive factors underlying these outcomes are not yet fully understood [7,8,9]. This knowledge gap is particularly important in Asian populations, who exhibit distinct obesity phenotypes compared with Western cohorts, including a higher proportion of visceral adiposity, greater insulin resistance, and earlier onset of metabolic complications at lower BMI levels. These differences have led to the adoption of lower BMI thresholds for bariatric surgery in Asian guidelines. Moreover, prior studies have suggested variations in metabolic remission rates and predictive factors between Asian and Western populations. Collectively, these population-specific characteristics underscore the need for dedicated analyses in Asian cohorts to better define metabolic outcomes and their predictors [10,11].

To enhance patient care and guide personalized treatment approaches, we evaluated metabolic resolution after bariatric surgery in Thai patients as an Asian population, identified predictors of favorable postoperative outcomes, and compared remission rates between SG and RYGB.

## 2. Materials and Methods

### 2.1. Study Design and Patient Selection

This retrospective study was conducted at a tertiary care hospital between January 2012 and December 2022 and included all individuals diagnosed with morbid obesity who underwent laparoscopic SG or RYGB at our institution. The choice of procedure was based on patient comorbidities, as detailed in Figure S1. Patients who underwent other bariatric procedures or those treated at other hospitals were excluded.

### 2.2. Definition of Diagnostic Criteria for Comorbidities

Metabolic remission was defined according to established international criteria for T2DM [12], HT [13], DLP [14], and MetS [5], as detailed in Table S1.

### 2.3. Data Collection

Patient characteristics, including age, sex, body mass index (BMI), and obesity-related comorbidities such as T2DM, DLP, HT, and MetS, were recorded. Preoperative medication use, including antidiabetic agents, lipid-lowering drugs, and antihypertensive medications, was also documented.

### 2.4. Surgical Procedures

SG involves vertical resection of the stomach from approximately 5–6 cm proximal to the pylorus to the angle of His using multiple linear staplers guided by a 36-French bougie tube.

In RYGB, 100 cm biliopancreatic and 100 cm alimentary limbs were created following an antecolic route. A small gastric pouch of approximately 20 cc was formed, and the pouch and jejunum were connected using a linear stapled technique, typically creating a gastrojejunostomy diameter of 2 cm.

### 2.5. Postoperative Follow-Up

The follow-up schedule included evaluations every 3 months for the first 18 months postoperatively, then every 6 months until the 5-year mark, and thereafter once a year. Laboratory assessments were conducted during each follow-up visit to evaluate the remission of comorbidities, including T2DM, DLP, HT, and MetS.

### 2.6. Definition of Remission or Resolution Criteria for the Comorbidities

Table S2 presents the definitions of remission criteria for each comorbidity. HT and DLP remission criteria were based on standardized outcomes in metabolic and bariatric surgery [14]. T2DM remission followed the criteria outlined in the Consensus Report [15], while MetS remission was defined according to the International Diabetes Federation [16].

### 2.7. Statistical Analysis

Descriptive statistics were used to summarize the patients’ characteristics and outcomes. Categorical variables were analyzed using the chi-square or Fisher’s exact test, whereas continuous variables were analyzed using Student’s *t*-test or the Mann–Whitney U test. Multivariate and Cox regression analyses were performed to adjust for confounding variables and identify independent predictors of remission. The Cox proportional hazards assumption was also tested. Survival analysis was performed using the Kaplan–Meier curve to estimate the time to remission. Predictors of remission were analyzed using Cox proportional hazards regression with backward stepwise regression. The cutoff points for continuous variables were determined using the Youden index with receiver operating characteristic curves unless otherwise indicated. Statistical analyses were performed using R software (version 4.1.1), and a *p*-value < 0.05 indicated statistical significance.

To minimize baseline differences between SG and RYGB groups, propensity score matching was performed using a nearest-neighbor approach without replacement, with a 1:1 matching ratio. Propensity scores were calculated based on sex, age, BMI category, and baseline comorbidities, including T2DM, HT, DLP, and MetS. A caliper width of 0.1 of the standard deviations of the logit of the propensity score was applied. Covariate balance before and after matching was assessed using standardized mean differences, with values < 0.1 indicating adequate balance.

## 3. Results

### 3.1. Overall Baseline Characteristics

This study included 581 patients, of whom 64.9% were women. The median age was 34.41 years (interquartile range [IQR], 27.3–42.7 years). The median BMI was 45.54 kg/m^2^ (IQR, 41.1–52.1 kg/m^2^), and the median body weight was 122.5 kg (IQR, 107.0–144.0 kg). At baseline, 191 individuals (32.9%) had T2DM, 264 (45.4%) had HT, 428 (73.7%) had DLP, and 458 (78.8%) had MetS. The median follow-up duration was 31 months. Follow-up rates were 85.7% (498/581) at 1 year and 46.6% (111/238) at 5 years. Baseline characteristics are summarized in Table 1.

### 3.2. Outcomes of Bariatric Surgery

#### 3.2.1. Weight Loss

**Figure 1:**
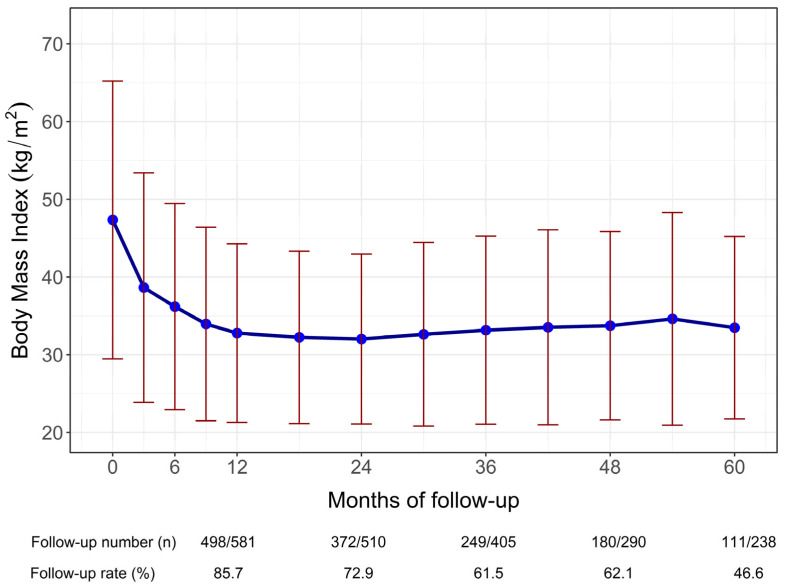
Trends in body mass index (BMI) over the follow-up period, with the blue line representing mean BMI values and the red line indicating values exceeding two standard deviations above the mean.

Following bariatric surgery, the mean (SD) initial BMI was 47.3 kg/m^2^ (8.9), which decreased to 32.8 (5.7) kg/m^2^ at 12 months, reaching the lowest BMI of 32.0 (5.5) kg/m^2^ at 24 months and 33.5 (5.9) kg/m^2^ at 60 months (Figure 1). The mean (SD) percentage total weight loss (%TWL) after 1–5 years of follow-up was 30.4 (7.9), 31.1 (9.7), 29.9 (10.9), 29.0 (10.2), and 28.0 (9.8), respectively.

#### 3.2.2. Cumulative Probability of Remission

For cumulative remission analyses, only patients with a confirmed diagnosis of each metabolic comorbidity at baseline who had complete follow-up data and sufficient information for remission assessment were included. Accordingly, cumulative remission was evaluated in patients with type 2 diabetes mellitus (T2DM; *n* = 154), hypertension (*n* = 162), dyslipidemia (*n* = 173), and metabolic syndrome (*n* = 407).

**Figure 2:**
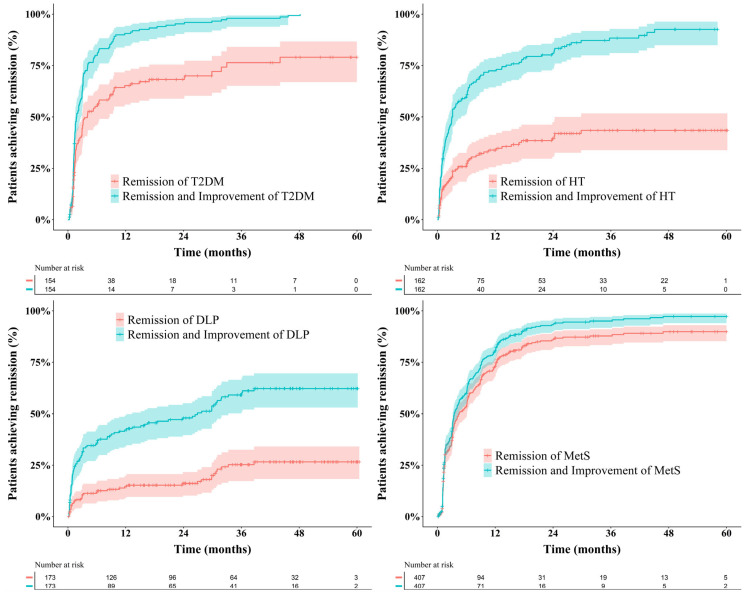
Trends in remission (red line) and combined rates of remission and improvement (green line) for comorbidities, including A. type 2 diabetes mellitus (T2DM), B. hypertension (HT), C. dyslipidemia (DLP), and D. metabolic syndrome (MetS), among all patients following bariatric surgery. DLP, dyslipidemia; HT, hypertension; MetS, metabolic syndrome; T2DM, type 2 diabetes mellitus.

Remission of T2DM was achieved in 97 patients (79.1%), while 150 patients (99.3%) experienced either remission or improvement. Dyslipidemia remission occurred in 59 patients (33.9%), with a combined remission and improvement rate of 55.2%. Hypertension remission was observed in 59 patients (36.0%), and 139 patients (84.8%) achieved remission or improvement. Remission of metabolic syndrome occurred in 362 patients (79.6%), with 402 patients (88.4%) demonstrating remission or improvement. The cumulative outcomes are illustrated in Figure 2.

### 3.3. Comparison of SG and RYGB After Propensity Score Matching

A total of 254 patients were included after propensity score matching, and their baseline characteristics are summarized in Table 2. The matched sleeve gastrectomy (SG) and Roux-en-Y gastric bypass (RYGB) groups were well balanced with respect to age, sex, body mass index, and the prevalence of metabolic comorbidities. In the propensity score–matched cohort, the median duration of T2DM was longer in the RYGB group than in the SG group (24.3 months [IQR 3.5–60.1] vs. 12.0 months [IQR 4.0–48.1]); however, this difference did not reach statistical significance (*p* = 0.370). Weight loss outcomes were comparable between SG and RYGB over 5 years. The mean (SD) %TWL for SG vs. RYGB at years 1–4 was 31.1 (8.0) vs. 31.7 (7.3), *p* = 0.61; 31.1 (9.2) vs. 32.1 (9.3), *p* = 0.53; 28.7 (9.6) vs. 31.8 (9.7), *p* = 0.12; and 30.2 (10.0) vs. 30.1 (10.0), *p* = 0.98, respectively. At 5 years, the median %TWL (IQR) was 29.8 (13.1) vs. 25.5 (14.1), *p* = 0.18.

**Figure 3:**
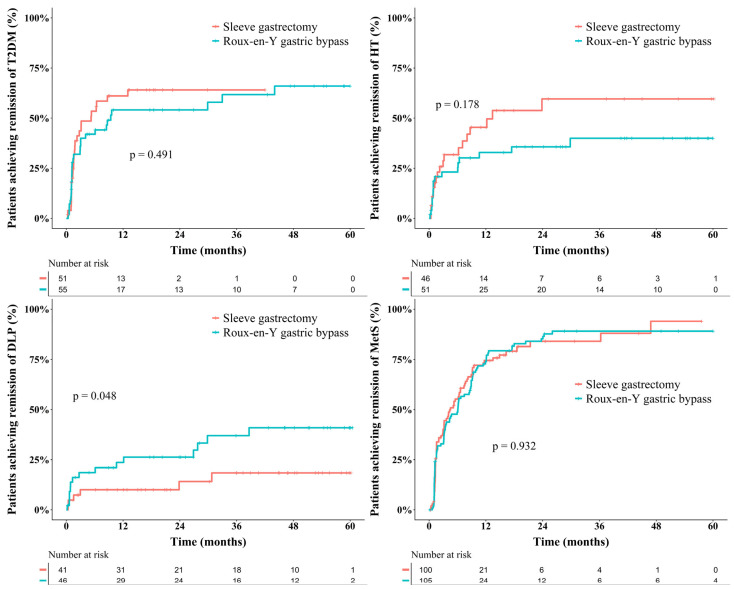
Cumulative remission trends after laparoscopic sleeve gastrectomy (LSG; red line) and laparoscopic Roux-en-Y gastric bypass (LRYGB; green line) for type 2 diabetes mellitus (T2DM), hypertension (HT), dyslipidemia (DLP), and metabolic syndrome (MetS) in propensity score-matched cohorts.

Cumulative remission outcomes are illustrated in Figure 3. At 3 years, the likelihood of T2DM remission was 64.1% (95% confidence interval [CI], 45.9–76.2%) after SG and 61.7% (95% CI, 42.9–74.4%) after RYGB (*p* = 0.491). The 4-year remission probabilities for HT were 59.6% (95% CI, 35.7–74.6%) with SG and 40.0% (95% CI, 22.1–53.7%) with RYGB (*p* = 0.178). At 5 years, DLP remission was significantly higher after RYGB (40.9%; 95% CI, 21.5–55.6%) than after SG (18.4%; 95% CI, 3.2–31.2%; *p* = 0.048). MetS showed the highest improvement, with 4-year remission estimates of 94.0% (95% CI, 70.4–98.8%) after SG and 89.1% (95% CI, 80.2–94.0%) after RYGB (*p* = 0.178), as shown in Table S3.

### 3.4. Factors Predicting Metabolic Remission

#### 3.4.1. T2DM

Univariate analysis showed significant differences between remission and non-remission groups in T2DM duration, preoperative HbA1c levels, fasting blood glucose (FBG) levels, and insulin dependence. Multivariate analysis revealed that only patients with T2DM for <3 years had a significantly higher remission rate (hazard ratio [HR] = 2.40, 95% CI, 1.41–4.07; *p* = 0.001) (Table 3).

#### 3.4.2. HT

Univariate analysis indicated younger age, fewer preoperative antihypertensive medications, and the presence of DLP as factors associated with HT remission. In multivariate analysis, age < 40 years (HR = 1.80, 95% CI, 1.04–3.12; *p* = 0.034) and the use of fewer than two antihypertensive drugs (HR = 0.62, 95% CI, 0.47–0.83; *p* < 0.001) remained significant predictors of remission (Table 4).

#### 3.4.3. DLP

Both univariate and multivariate analyses indicated age < 44 years (Adjusted HR = 2.16, 95% CI, 1.03–4.50; *p* = 0.032) and undergoing RYGB (HR = 2.12, 95% CI, 1.09–4.12; *p* = 0.032) as independent predictors of remission (Table 5).

#### 3.4.4. MetS

In the univariate analysis, male sex, lower preoperative BMI, and lower preoperative FBG and HbA1c levels differed significantly between remission and non-remission groups. Multivariate analysis confirmed male sex (HR = 0.78, 95% CI, 0.61–0.98; *p* = 0.034), preoperative BMI < 43 kg/m^2^ (HR = 1.34, 95% CI, 1.07–1.68; *p* = 0.013), and FBG level < 126 mg/dL (HR = 1.43, 95% CI, 1.08–1.88; *p* = 0.009) as predictors of remission (Table 6).

## 4. Discussion

### 4.1. Baseline Characteristics

Most participants in this study were women, a pattern consistent with previous reports [6,17]. Despite the relatively young mean age of 35.9 years, our cohort’s age distribution aligns with earlier studies reporting mean ages between 34.7 and 43.2 years [6,18]. This trend reflects the growing burden of obesity among younger populations. Although indications for bariatric surgery are generally lower in Asian candidates than in Western patients [19,20], our cohort had a considerably higher mean BMI (47.3 kg/m^2^), exceeding values reported in Western (40.3–44.0 kg/m^2^) and non-Western (37.4–43.3 kg/m^2^) studies [21,22]. This underscores the severity of obesity in young Thai patients, especially given the high prevalence of comorbidities such as obstructive sleep apnea (OSA), MetS, DLP, HT, T2DM, and fatty liver disease.

Although BMI was high, most patients (78.1%) underwent SG, consistent with prior reports [6,18]. RYGB was performed in fewer patients (21.9%), yet metabolic outcomes remained satisfactory. The median follow-up duration of 2.6 years was similar to that reported in other studies [18,23].

### 4.2. Outcomes of Bariatric Surgery

#### 4.2.1. Weight Loss and Metabolic Resolution

Weight loss following bariatric surgery typically follows a characteristic trajectory, with rapid reduction during the first postoperative year, reaching a nadir within 1–2 years, followed by partial weight regain and subsequent long-term stabilization. This pattern has been consistently reported in large longitudinal cohorts, including the Swedish Obese Subjects (SOS) study [24], which demonstrated sustained weight loss and metabolic benefits despite modest weight regain over extended follow-up. Similar weight loss trajectories have also been observed in Asian populations, reflecting shared physiological responses to bariatric procedures across ethnic groups [25,26]. A comparable pattern was observed in our cohort, as depicted in Figure 1.

Trends in body mass index over time suggested partial weight regain after the initial postoperative nadir, particularly beyond two years of follow-up. Weight regain is a recognized phenomenon after bariatric surgery and may influence the durability of metabolic remission [27]. This observation highlights the importance of long-term follow-up, lifestyle modification, and procedure selection tailored to patients at higher risk of weight recurrence. Further studies with extended follow-up are needed to clarify the relationship between weight trajectories and long-term metabolic outcomes.

In our study, weight loss outcomes were comparable between SG and RYGB, with an overall mean %TWL of 30.4% at 1 year and 28.0% at 5 years. Consistent with these findings, previous studies in Asian populations have reported %TWL values of 30.2–32.4% at 1 year [19,22] and 23.7–29.5% at 5 years after SG [22,28]. Similar outcomes have also been reported after RYGB, with %TWL values of 29.5% at 1 year and 28.1% at 5 years [29].

In addition to significant weight loss, bariatric surgery in our cohort resulted in remission of multiple metabolic comorbidities, consistent with previous reports. Sustained weight reduction after SG and RYGB has been shown to improve or resolve T2DM, HT, DLP, and MetS [6,30,31,32,33,34,35]. The following sections examine remission outcomes for each condition in detail.

#### 4.2.2. T2DM

The T2DM remission rate was 79.1%, consistent with long-term studies reporting remission rates of 57–68% [4,36]. Most remissions occur within the first year, reflecting both weight loss-dependent and weight loss-independent mechanisms. Early glycemic improvement is driven by acute caloric restriction, reduced hepatic insulin resistance, and enhanced incretin responses (particularly GLP-1), while sustained weight loss in subsequent years further improves peripheral insulin sensitivity [37,38]. Procedure-specific changes in bile acid signaling and gut hormone secretion may also contribute to durable glycemic control, especially after bypass procedures [39].

Shorter T2DM duration was the strongest predictor of remission, aligning with earlier findings [40,41]. Mechanistically, shorter disease duration likely reflects better preserved pancreatic β-cell reserve and less irreversible β-cell dysfunction, thereby increasing the likelihood of postoperative glycemic normalization. This concept is supported by prediction models developed and validated in Asian populations, such as the ABCD score (age, BMI, C-peptide, and diabetes duration), which emphasizes preserved β-cell function as a key determinant of remission [42,43]. The 36-month cutoff in this study demonstrated strong predictive value, while other studies reported thresholds between 1 and 5 years [44,45]. Hage et al. reported an estimated 7% reduction in remission probability for each additional year of diabetes duration, reinforcing the benefit of earlier surgical intervention [46].

After propensity score matching, no statistically significant difference in T2DM remission was observed between the SG and RYGB groups, despite prior studies including those from Asian cohorts reporting superior outcomes with RYGB [30,32]. However, some studies from Asian cohorts have suggested no clear difference in remission between SG and RYGB, indicating that procedure-specific advantages may be less pronounced in certain populations [17,47]. In our study, the Kaplan–Meier curves demonstrated a numerically higher remission rate following SG; however, this trend should be interpreted cautiously and not as evidence of superiority. Residual differences in unmeasured factors—such as baseline β-cell reserve (e.g., C-peptide), heterogeneity in disease severity, postoperative behaviors, and medication adjustments—may have influenced remission trajectories [40,41,46].

#### 4.2.3. HT

HT remission was 36.0%, lower than the previously reported range of 42–68% [48,49]. However, when improvement was included, over 84% benefited, consistent with prior studies [36,50]. This distinction between complete remission and partial improvement is particularly relevant in cohorts with severe obesity and long-standing disease, where full normalization of blood pressure may be biologically less achievable [51,52]. Patients in our cohort began with markedly elevated BMIs, and many remained within the obese range despite significant loss, suggesting persistent cardiometabolic burden that may limit complete HT resolution.

Several mechanistic pathways may explain the relatively modest remission rate. Although bariatric surgery induces substantial weight loss, residual visceral adiposity can continue to promote sympathetic nervous system activation, renin–angiotensin–aldosterone system (RAAS) upregulation, endothelial dysfunction, and increased arterial stiffness [52,53]—key contributors to obesity-related hypertension. In patients with prolonged disease duration, structural vascular remodeling and reduced arterial compliance may become irreversible [53,54], thereby attenuating the likelihood of full remission despite improved blood pressure control.

Several confounding factors, including dietary salt intake, medication adherence, smoking status, alcohol use, OSA severity, CPAP efficacy, nocturnal dipping, and physical activity, were not fully evaluated [55]. These unmeasured variables may partly account for inter-study heterogeneity in HT outcomes and underscore the multifactorial nature of post-bariatric blood pressure regulation [51]. Consistent with previous reports, younger age and fewer preoperative antihypertensive medications were significant predictors of HT remission [56,57]. Younger patients are more likely to exhibit functional rather than fixed vascular abnormalities [53], whereas long-standing hypertension is associated with progressive arterial stiffening and microvascular damage. Accordingly, earlier surgical intervention may mitigate irreversible vascular injury and improve the probability of complete remission [58,59,60].

When contextualized within the Asian literature, our findings are broadly consistent with prior regional cohort studies, which generally report lower HT remission rates compared with Western populations [61,62] despite substantial metabolic improvement. Asian patients tend to develop hypertension at lower BMI thresholds and often demonstrate greater salt sensitivity [10,63], higher visceral fat accumulation, and distinct genetic and environmental risk profiles. These population-specific characteristics may blunt the antihypertensive effects of weight loss alone, rendering improvement—rather than complete remission—a more clinically meaningful endpoint in Asian cohorts [51].

Matched analysis revealed no significant difference in HT remission between SG and RYGB, consistent with previous reports [30,32]. Although some reports suggest higher long-term remission following RYGB at five years [30], our findings indicate that baseline disease severity, age, and antihypertensive burden may exert a greater influence on HT outcomes than procedure-specific hormonal mechanisms [51,64]. This observation supports the concept that patient selection and timing of intervention may be more critical determinants of HT remission than the choice of bariatric procedure itself.

#### 4.2.4. DLP

DLP affected nearly three-quarters of our cohort, with a remission rate of 33.9%. This aligns with the findings of Coleman et al. [31], who reported remission rates of 28% after SG and 38% after RYGB at 4 years. Other studies have reported higher remission rates (43–50%), which may be attributable to differences in remission definitions [23,50]. Shah et al. [65] reported an 89% remission rate based solely on high-density lipoprotein cholesterol and triglycerides. In this study, younger age (<44 years) and undergoing RYGB were strong predictors of remission, consistent with previous findings [66].

Regarding lipid profiles, triglyceride levels decreased significantly, and HDL levels increased. In contrast, total cholesterol and LDL levels showed no significant change, consistent with earlier reports [67]. RYGB was more effective than SG in achieving DLP remission, echoing prior observations [68,69]. This effect may be explained by altered bile acid circulation after RYGB. Such changes reduce LDL reabsorption in a manner similar to ileal bypass [70]. Previous studies demonstrated a 33% reduction in LDL-C after ileal bypass, whereas our study showed a 17% reduction following RYGB (Table S4). Taken together, these findings suggest that RYGB may be the preferred procedure for achieving dyslipidemia remission.

Beyond changes in serum lipid levels, Roux-en-Y gastric bypass is associated with procedure-specific metabolic effects that may further explain its superiority in dyslipidemia remission [71]. These include enhanced bile acid signaling through activation of farnesoid X receptor and TGR5 pathways, reduced intestinal lipid absorption, and alterations in gut hormone secretion, all of which contribute to improved cholesterol homeostasis [72]. Such mechanisms are less pronounced after sleeve gastrectomy, where metabolic improvements are more closely linked to weight loss-dependent effects [73]. While improved lipid profiles may suggest cardiovascular benefit [59,74], long-term cardiovascular outcome data were not assessed in this study.

#### 4.2.5. MetS

MetS remission was 79.6%, consistent with prior reports of 82–86.2% [65,75]. Weight loss, particularly reduction in visceral fat, is central to improvement [76]. Male sex, BMI < 43 kg/m^2^, and FBG level < 126 mg/dL predicted remission in our study. Men may experience greater remission due to proportionally higher visceral fat loss and hormonal differences, particularly increases in testosterone [77,78]. However, prior reports are mixed, with some demonstrating higher remission among women [79] and others showing no significant sex differences [76]. The smaller number of men in our study may partly explain the observed outcomes. Lower preoperative BMI was also associated with higher remission, likely because patients with less severe metabolic disturbances have a greater chance of resolution [51,80]. Similarly, a preoperative FBG level below 126 mg/dL may represent milder dysglycemia within the spectrum of metabolic syndrome rather than established diabetes [81,82]. Because MetS is defined by a constellation of metabolic abnormalities, better baseline glycemic status may indicate less advanced metabolic derangements and, consequently, a higher probability of achieving remission after surgery, consistent with prior findings [51,83]. Although direct comparative evidence between SG and RYGB remains limited, our study indicates no significant difference in MetS remission between the two procedures [64].

From a regional perspective, our MetS remission rate is comparable to those reported in Asian cohorts, which generally demonstrate high rates of metabolic improvement despite lower baseline BMI compared with Western populations. Prior Asian studies have shown that reductions in visceral adiposity and early-stage glycemic dysregulation are key determinants of MetS resolution following bariatric surgery, supporting the relevance of baseline metabolic severity in this population [61,84].

### 4.3. Strengths and Limitations

The present study included a relatively large cohort with long-term follow-up ranging from 1 to 5 years (median 2.6 years). At 5 years, nearly half of the initial cohort remained in follow-up, while overall adherence was maintained at approximately 75% through telecommunication support and referral to local hospitals. The large sample size enhanced statistical power and reduced heterogeneity, despite being derived from a single institution. Baseline characteristics were compared between patients who continued follow-up and those lost to follow-up. No major differences were observed (Table S5), supporting the representativeness of the analyzed cohort. Nevertheless, several important limitations should be acknowledged. First, although baseline characteristics were comparable, outcomes among patients lost to follow-up could not be directly assessed. Substantial attrition at five years may introduce attrition bias and reduce the precision of long-term remission estimates, particularly for chronic metabolic outcomes. In contrast, previous reports have shown substantially lower follow-up adherence, decreasing to 30% by the second year and falling below 10% by the tenth year [85]. In addition, as discussed earlier, postoperative weight regain was not systematically evaluated in this study, which may have influenced long-term weight trajectories and the durability of metabolic remission. Second, the retrospective design of this study relied on existing medical records, which limited control over potential confounding variables and precluded standardized assessment of all relevant exposures. Third, several potentially important confounders, including dietary patterns, physical activity levels, and socioeconomic status, were not systematically captured and may have influenced long-term metabolic outcomes. The cohort, comprising young individuals with severe obesity, illustrates the substantial burden of metabolic comorbidities associated with the rising prevalence of obesity in younger populations. These factors should be considered when interpreting the results.

Although the large sample size and use of propensity score matching helped mitigate measured confounding, residual confounding cannot be excluded. Finally, as this was a single-center study, the generalizability of the findings to other populations and healthcare settings may be limited. However, the homogeneity of surgical techniques, perioperative care, and follow-up protocols strengthened internal validity and reduced procedural heterogeneity. Taken together, these strengths and limitations underscore the need for cautious interpretation of long-term remission outcomes and highlight the value of future prospective, multicenter studies with standardized assessment of lifestyle and socioeconomic factors.

## 5. Conclusions

Bariatric surgery effectively improved metabolic comorbidities, with several predictive factors identified for remission. Although overall outcomes of SG and RYGB were comparable, RYGB provided superior remission of dyslipidemia. These findings contribute to the limited long-term metabolic evidence available from Asian populations and may support clinical decision-making and healthcare planning in similar settings.

## Figures and Tables

**Table 1:** Patient characteristics (*n* = 581).

Characteristic	Total (*n* = 581)
Age (years), median (IQR) <30 30–39 40–49 50–59 ≥60	34.4 (27.3, 42.8) 201 (34.6) 199 (34.3) 102 (17.6) 63 (10.8) 16 (2.8)
Female sex, *n* (%)	377 (64.9)
Body weight (kg), median (IQR)	122.5 (107.0, 144.0)
BMI (kg/m^2^), median (IQR)	45.5 (41.1, 52.1)
<40	123 (21.2)
40–50	287 (49.4)
>50	171 (29.4)
Comorbidity, *n* (%): Type 2 diabetes mellitus	191 (32.9)
Insulin dependent Duration of type 2 diabetes mellitus (months), median (IQR)	30 (5.2) 12.0 (2.1, 48.6)
Hypertension	264 (45.4)
Dyslipidemia	428 (73.7)
Metabolic syndrome	458 (78.8)
Fatty liver, *n* (%)	527 (90.7)
Obstructive sleep apnea, *n* (%) Mild Moderate Severe Primary snoring	112 (19.5) 106 (18.4) 289 (50.3) 68 (11.8)
Gastroesophageal reflux disease, *n* (%)	71 (12.2)
Number of drugs, *n* (%)	
Oral antidiabetic medication 0 1 2 3	56 (29.5) 79 (41.6) 37 (19.5) 18 (9.5)
Anti-hypertensive medication 0 1 2 ≥3	57 (21.8) 88 (33.7) 63 (24.1) 53 (20.3)
Lipid-lowering medication 0 1 2	261 (61.1) 163 (38.2) 3 (0.7)
Relevant biochemical study and parameter	
Type 2 diabetes mellitus (*n* = 191)	
Fasting blood glucose (mg/dL), median (IQR) HOMA-IR ^a^, median (IQR) HOMA-B ^b^, median (IQR) Hemoglobin A1c (%), median (IQR) <6.5 6.5–7.9 ≥8.0	124.5 (109.8, 153.0) 10.3 (6.3, 15.1) 4.8 (3.1, 7.4) 6.9 (6.4, 8.1) 48 (25.3) 90 (47.4) 52 (27.4)
Blood pressure (*n* = 264)	
Systolic blood pressure (mmHg), mean (SD) Diastolic blood pressure (mmHg), mean (SD)	143.4 (17.3) 85.4 (12.0)
Dyslipidemia (*n* = 428)	
LDL-C, (mg/dL), median (IQR) HDL-C (mg/dL), median (IQR) Triglycerides (mg/dL), median (IQR) Cholesterol, median (IQR)	133.2 (108.1, 165.6) 40.7 (35.6, 48.3) 135.0 (103.0, 181.2) 188.5 (160.0, 223.0)
Operation Sleeve gastrectomy Roux-en-Y gastric bypass	454 (78.1%) 127 (21.9%)

Data are presented as *n* (%) unless otherwise indicated. ^a^ HOMA-IR: Homeostasis Model Assessment of Insulin Resistance; HOMA-IR = (Fasting insulin level in μIU/mL) × (Fasting glucose level in mg/dL)/405. ^b^ HOMA-B: Homeostasis Model Assessment of Beta-cell Function; HOMA-B = (Fasting insulin level in μIU/mL) × 20/(Fasting glucose level in mg/dL—3.5). BMI, body mass index; CI, confidence interval; HDL-C, high-density lipoprotein cholesterol; IQR, interquartile range; LDL-C, low-density lipoprotein cholesterol; SD, standard deviation.

**Table 2:** Patient characteristics after propensity score matching (*n* = 254).

Characteristic	Sleeve Gastrectomy (*n* = 127)	Roux-en-Y Gastric Bypass (*n* = 127)	*p*-Value
Age (years), median (IQR) <30 30–39 40–49 50–59 ≥60	35.8 (30.4, 41.5) 29 (22.8) 58 (45.7) 28 (22.0) 10 (7.9) 2 (1.6)	36.2 (30.3, 43.0) 30 (23.6) 54 (42.5) 28 (22.0) 13 (10.2) 2 (1.6)	0.613 0.968
Female sex, *n* (%)	90 (70.9)	89 (70.1)	1.000
Body weight (kg), median (IQR)	125.6 (108.0, 144.0)	123.0 (107.3, 145.6)	0.813
BMI (kg/m^2^), median (IQR)	45.5 (41.0, 55.0)	46.6 (41.8, 54.8)	0.782
<40	25 (19.7)	23 (18.1)	0.917
40–50	56 (44.1)	59 (46.5)	
>50	46 (36.2)	45 (35.4)	
Comorbidity, *n* (%) Type 2 diabetes mellitus	66 (52.0)	67 (52.8)	1.000
Insulin dependence	13 (10.2)	17 (13.4)	0.730
Hypertension	76 (59.8)	72 (56.7)	0.703
Dyslipidemia	94 (74.0)	92 (72.4)	0.887
Metabolic syndrome	111 (87.4)	112 (88.2)	1.000

Data are presented as *n* (%) unless otherwise indicated. BMI, body mass index; IQR, interquartile range.

**Table 3:** Predictors of remission in patients with type 2 diabetes mellitus 5 years after bariatric surgery.

Variable	Remission Group	No Remission Group	^a^ Crude HR (95% CI)	*p*-Value	^b^ Adjusted HR (95% CI)	*p*-Value
Remission of type 2 diabetes mellitus (*n* = 154)	97 (63.0)	57 (37.0)				
Age (years): <35	35 (36.1)	22 (38.6)	1.19 (0.77–1.84)	0.424		
Sex: male vs. female	35 (36.1)	21 (36.8)	1.16 (0.75–1.81)	0.498		
Duration of diabetes mellitus (years): <3 years	69 (75.8)	25 (46.3)	2.88 (1.75–4.74)	<0.001	2.40 (1.41–4.07)	0.001
Operation: RYGB vs. SG	30 (30.9)	25 (43.9)	0.59 (0.37–0.95)	0.030		
Preoperative BMI (kg/m^2^): <45	46 (47.4)	32 (56.1)	0.86 (0.56–1.3)	0.468		
Preoperative HbA1c (mg%): <7	55 (57.3)	23 (40.4)	1.64 (1.07–2.51)	0.023		
Drug: insulin users vs. nonusers	10 (10.3)	15 (26.3)	0.46 (0.24–0.89)	0.021		
FBG (mg/dL): <126	53 (54.6)	24 (42.1)	1.60 (1.05–2.44)	0.030		
Preoperative insulin use (cont.): median (IQR)	33.9 (20.8, 50.4)	29 (20.7, 45.2)	0.99 (0.99–1.00)	0.951		

Data are presented as *n* (%) unless otherwise indicated. ^a^ Univariate Cox proportional hazard regression analysis. ^b^ Multivariate Cox proportional hazard regression analysis. BMI, body mass index; CI, confidence interval; HbA1c, hemoglobin A1c; HR, hazard ratio; IQR, interquartile range; RYGB, Roux-en-Y gastric bypass; SG, sleeve gastrectomy; vs., versus.

**Table 4:** Predictors of remission in patients with hypertension 5 years after bariatric surgery.

Variable	Remission Group	No Remission Group	^a^ Crude HR (95% CI)	*p*-Value	^b^ Adjusted HR (95% CI)	*p*-Value
Hypertension remission (*n* = 162)	58 (35.8)	104 (64.2)				
Age (year): <40	34 (58.6)	43 (41.3)	2.09 (1.23–3.53)	0.006	1.80 (1.04–3.12)	0.034
Sex: male vs. Female	20 (34.5)	38 (36.5)	1.13 (0.66–1.94)	0.664		
Operative procedure: RYGB vs. SG	17 (29.3)	34 (32.7)	0.89 (0.51–1.57)	0.693		
Preoperative BMI (kg/m^2^): <35	2 (3.4)	3 (2.9)	1.05 (0.26–4.31)	0.945		
Preoperative number of anti-hypertensive medications, median (IQR)	1 (1, 2)	2 (1, 3)	0.61 (0.45–0.81)	<0.001	0.62 (0.47–0.83)	<0.001
Obstructive sleep apnea: ref. = snoring	6 (10.3)	5 (4.8)	ref.			
Mild	5 (8.6)	14 (13.5)	0.43 (0.13–1.41)	0.164		
Moderate	13 (22.4)	16 (15.4)	0.62 (0.23–1.63)	0.330		
Severe	34 (58.6)	69 (66.3)	0.46 (0.19–1.10)	0.080		
Cardiovascular disease	4 (6.9)	19 (18.3)	0.43 (0.16–1.19)	0.105		
Type 2 diabetes mellitus	30 (51.7)	58 (55.8)	1.02 (0.61–1.71)	0.946		
Dyslipidemia	44 (75.9)	93 (89.4)	0.50 (0.27–0.91)	0.023		

Data are presented as *n* (%) unless otherwise indicated. ^a^ Univariate Cox proportional hazard regression analysis. ^b^ Multivariate Cox proportional hazard regression analysis. BMI, body mass index; CI, confidence interval; HR, hazard ratio; IQR, interquartile range; ref., reference; RYGB, Roux-en-Y gastric bypass; SG, sleeve gastrectomy; vs., versus.

**Table 5:** Predictors of remission in patients with dyslipidemia 5 years after bariatric surgery.

Variable	Remission Group	No Remission Group	^a^ Crude HR (95% CI)	*p*-Value	^b^ Adjusted HR (95% CI)	*p*-Value
Dyslipidemia remission (*n* = 173)	36 (20.8)	137 (79.2)				
Age (year): <44	26 (72.2)	82 (59.9)	2.28 (1.10–4.75)	0.027	2.16 (1.03–4.50)	0.032
Sex: male vs. female	13 (36.1)	49 (35.8)	1.12 (0.57–2.21)	0.745		
Operative procedure: RYGB vs. SG	15 (41.7)	31 (22.6)	2.26 (1.16–4.38)	0.016	2.12 (1.09–4.12)	0.032
Preoperative BMI (kg/m^2^): <43	17 (47.2)	60 (43.8)	1.08 (0.56–2.08)	0.820		
Preoperative lipid-lowering agent use, median (IQR)	1 (1, 1)	1 (0, 1)	1.91 (0.92–3.96)	0.084		
Obstructive sleep apnea: ref. = snoring	1 (2.8)	12 (8.8)	ref.			
Mild	7 (19.4)	26 (19.0)	3.42 (0.42–27.80)	0.251		
Moderate	8 (22.2)	15 (10.9)	6.40 (0.80–51.32)	0.081		
Severe	20 (55.6)	84 (61.3)	2.99 (0.40–22.31)	0.285		
Cardiovascular disease	2 (5.6)	16 (11.7)	0.49 (0.12–2.04)	0.326		
Type 2 diabetes mellitus	21 (58.3)	78 (56.9)	1.03 (0.53–2.00)	0.931		
Hypertension	27 (75.0)	88 (64.2)	1.47 (0.69–3.12)	0.321		
Chronic kidney disease	1 (2.8)	4 (2.9)	1.06 (0.14–7.73)	0.957		

Data are presented as *n* (%) unless otherwise indicated. ^a^ Univariate Cox proportional hazard regression analysis. ^b^ Multivariate Cox proportional hazard regression analysis. BMI, body mass index; CI, confidence interval; HR, hazard ratio; IQR, interquartile range; ref., reference; RYGB, Roux-en-Y gastric bypass; SG, sleeve gastrectomy; vs., versus.

**Table 6:** Predictors of remission in patients with metabolic syndrome 5 years after bariatric surgery.

Variable	Remission Group	No Remission Group	^a^ Crude HR (95% CI)	*p*-Value	^b^ Adjusted HR (95% CI)	*p*-Value
Metabolic syndrome remission (*n* = 407)	327 (80.3)	80 (19.7)				
Age (year): <40	222 (67.9)	53 (66.2)	1.02 (0.80–1.28)	0.896		
Sex: male vs. female	102 (31.2)	35 (43.8)	0.78 (0.62–0.99)	0.037	0.78 (0.61–0.98)	0.034
Operative procedure: RYGB vs. SG	88 (26.9)	17 (21.2)	0.98 (0.77–1.25)	0.862		
Preoperative BMI (kg/m^2^): <43	124 (37.9)	20 (25.0)	1.34 (1.07–1.67)	0.012	1.34 (1.07–1.68)	0.013
Obstructive sleep apnea: ref. = snoring	31 (9.5)	7 (8.8)	ref.			
Mild	63 (19.3)	16 (20.0)	1.01 (0.66–1.55)	0.970		
Moderate	68 (20.8)	12 (15.0)	1.1 (0.72–1.69)	0.650		
Severe	165 (50.5)	45 (56.2)	0.87 (0.59–1.28)	0.479		
Cardiovascular disease	24 (7.3)	6 (7.5)	1.01 (0.67–1.53)	0.968		
Type 2 diabetes mellitus	135 (41.3)	37 (46.2)	0.94 (0.75–1.17)	0.555		
Hypertension	162 (49.5)	40 (50.0)	0.99 (0.80–1.23)	0.921		
Dyslipidemia	252 (77.1)	68 (85.0)	0.87 (0.67–1.12)	0.285		
Chronic kidney disease	4 (1.2)	3 (3.8)	0.64 (0.24–1.70)	0.368		
Baseline level of TG (mg/dL): ≥150	127 (38.8)	36 (45.0)	0.88 (0.71–1.10)	0.270		
Fasting blood glucose (mg/dL): <126	263 (80.4)	57 (71.2)	1.36 (1.04–1.79)	0.027	1.43 (1.08–1.88)	0.009
Preoperative HbA1c (mg%): <7.0	264 (80.7)	54 (67.5)	1.34 (1.02–1.77)	0.036		
HOMA-IR: ≥8.0	147 (45.0)	49 (61.3)	0.86 (0.69–1.06)	0.160		

Data are presented as *n* (%) unless otherwise indicated. ^a^ Univariate Cox proportional hazard regression analysis. ^b^ Multivariate Cox proportional hazard regression analysis. BMI, body mass index; CI, confidence interval; HbA1c, hemoglobin A1c; HR, hazard ratio; HOMA-IR: Homeostasis Model Assessment of Insulin Resistance; HOMA-IR = (Fasting insulin level in μIU/mL) × (Fasting glucose level in mg/dL) / 405; IQR, interquartile range; RYGB, Roux-en-Y gastric bypass; SG, sleeve gastrectomy; TG, triglycerides.

## Data Availability

The data are not publicly available due to privacy and ethical restrictions involving patient medical records.

## Supplementary Materials

The following supporting information can be downloaded at: https://www.mdpi.com/article/10.3390/jcm15041539/s1, Figure S1: Types of bariatric surgery procedures and guidelines for procedure selection; Table S1: Definitions of the diagnostic criteria for each comorbidity; Table S2: Definitions of the remission criteria for each comorbidity; Table S3: Probability of remission outcomes by comorbidity and procedure; Table S4: Comparison of lipid profile variables before and after bariatric surgery; Table S5: Comparison of characteristics between patients who were lost to follow-up and those who continued follow-up at 5 years after bariatric surgery (*n* = 238).

## Abbreviations

The following abbreviations are used in this manuscript:

BMI	Body mass index
CI	Confidence interval
CVD	Cardiovascular disease
DLP	Dyslipidemia
FBG	Fasting blood glucose
HbA1c	Hemoglobin A1c
HOMA-IR	Homeostasis Model Assessment of Insulin Resistance
HR	Hazard ratio
HT	Hypertension
IQR	Interquartile range
MetS	Metabolic syndrome
OSA	Obstructive sleep apnea
RYGB	Roux-en-Y gastric bypass
SG	Sleeve gastrectomy
T2DM	Type 2 diabetes mellitus
TWL	Total weight loss
