# Supplementary material for: Long-Term Metabolic Remission and Predictive Factors After Sleeve Gastrectomy and Roux-en-Y Gastric Bypass in an Asian Population
Source: J Clin Med. 2026 Feb 15;15(4):1539. doi: 10.3390/jcm15041539 (PMC12942549; doi:10.3390/jcm15041539)
Supplement: Supplementary file 1 [file jcm-15-01539-s001.zip › Figure S1_181268.pdf]

Figure S1. Types of bariatric surgery procedures and guidelines for procedure selection

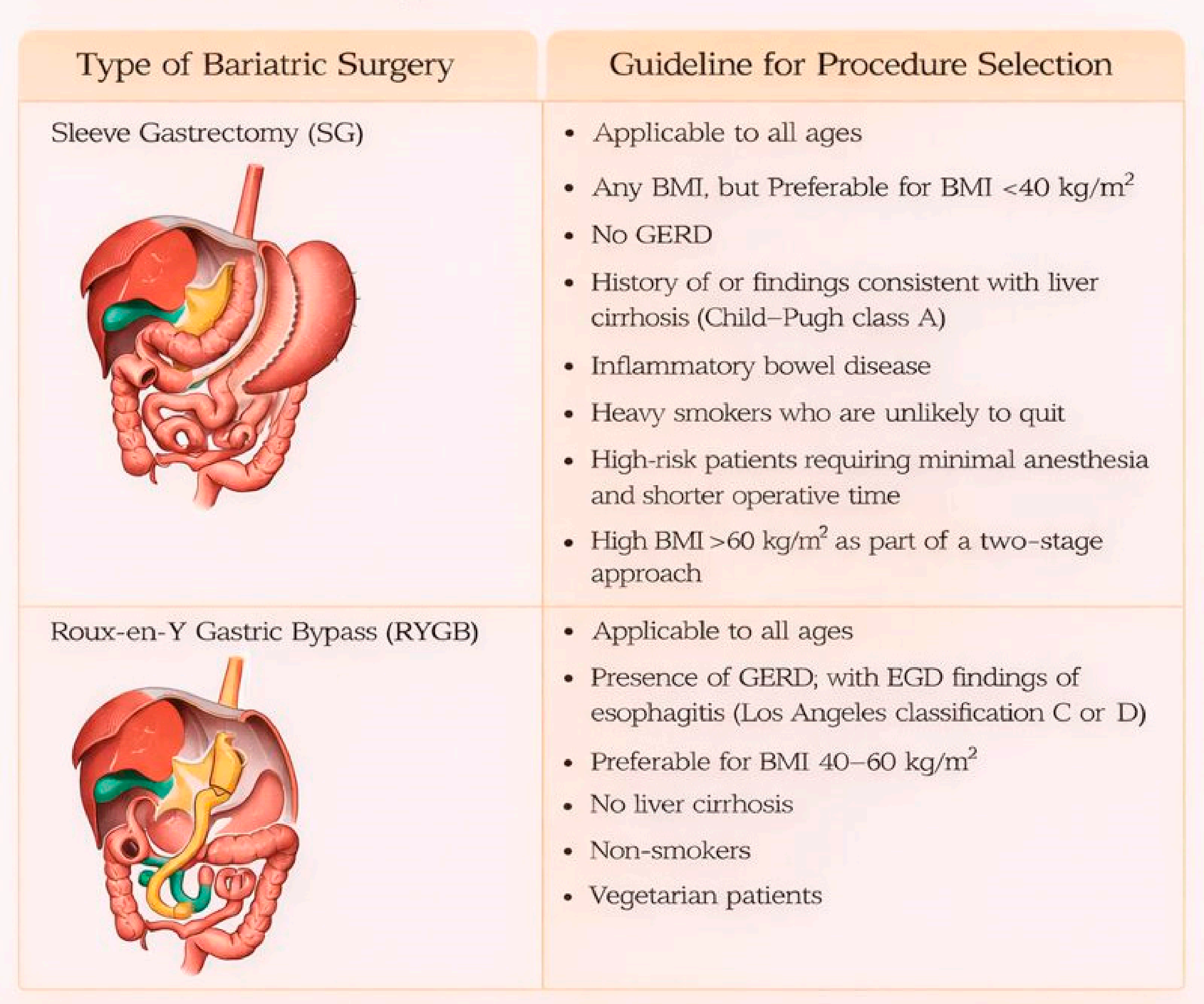

Type of Bariatric Surgery	Guideline for Procedure Selection
Sleeve Gastrectomy (SG)	• Applicable to all ages<br>• Any BMI, but Preferable for BMI <40 kg/m^2<br>• No GERD<br>• History of or findings consistent with liver cirrhosis (Child–Pugh class A)<br>• Inflammatory bowel disease<br>• Heavy smokers who are unlikely to quit<br>• High-risk patients requiring minimal anesthesia and shorter operative time<br>• High BMI >60 kg/m^2 as part of a two-stage approach
Roux-en-Y Gastric Bypass (RYGB)	• Applicable to all ages<br>• Presence of GERD; with EGD findings of esophagitis (Los Angeles classification C or D)<br>• Preferable for BMI 40–60 kg/m^2<br>• No liver cirrhosis<br>• Non-smokers<br>• Vegetarian patients

SG: Sleeve Gastrectomy; RYGB: Roux-en-Y Gastric Bypass
EGD: Esophagogastroduodenoscopy; GERD: Gastroesophageal Reflux Disease
