# Supplementary material for: Long-Term Metabolic Remission and Predictive Factors After Sleeve Gastrectomy and Roux-en-Y Gastric Bypass in an Asian Population
Source: J Clin Med. 2026 Feb 15;15(4):1539. doi: 10.3390/jcm15041539 (PMC12942549; doi:10.3390/jcm15041539)
Supplement: Supplementary file 1 [file jcm-15-01539-s001.zip › Table S1_181268.pdf]

Table S1. Definitions of the diagnostic criteria for each comorbidity

Disease	Diagnostic criteria	Reference
1. Hypertension	Systolic blood pressure ≥140 mm Hg or diastolic blood pressure ≥90 mm Hg	The 2018 European Society of Cardiology/European Society of Hypertension guideline.<br>(Eur Heart J. 2018;39(33):3021-104.)
2. T2DM	1. A1C ≥6.5%. The test should be performed in a laboratory using an NGSP-certified method standardized to the DCCT assay.*<br>OR<br>2. FPG level ≥126 mg/dL (7 mmol/L). Fasting is defined as no caloric intake for at least 8 h.*<br>OR<br>3. 2-h plasma glucose level ≥200 mg/dL (11.1 mmol/L) during an OGTT. The test should be performed as described by the World Health Organization, using a glucose load containing the equivalent of 75 g anhydrous glucose dissolved in water.*<br>OR<br>4. In a patient with classic symptoms of hyperglycemia or hyperglycemic crisis, a random plasma glucose level ≥200 mg/dL (11.1 mmol/L).<br>* In the absence of unequivocal hyperglycemia, diagnosis requires two abnormal test results from the same sample or in two separate test samples	American Diabetes Association criteria for the diagnosis of diabetes<br>(Diabetes Care. 2023;46(Suppl 1):S19-S40.)
3. Dyslipidemia	LDL 130–159 mg/dL = borderline high<br>LDL ≥160 mg/dL = high<br>HDL <40 mg/dL = low<br>HDL >60 mg/dL = high<br>Total cholesterol 200–239 = borderline high<br>Total cholesterol ≥240 mg/dL = high<br>TG 150–199 = borderline high<br>TG ≥200 mg/dL = high	Standardized outcomes reporting in metabolic and bariatric surgery<br>(Surg Obes Relat Dis. 2015;11(3):489-506.)
4. Metabolic syndrome	Increased waist circumference; men ≥90 cm, women ≥80 cm along with any two of the following features:<br>- Triglyceride ≥1.7 mmol/L (≥150 mg/dL) or TG treatment	International Diabetes Federation (IDF) 2009<br>(J Clin Med. 2020;9(12).)

	- HDL-C men <1.03 mmol/L (<40 mg/dl) or women <1.29 mmol/L (< 50 mg/dL) or HDL-C treatment<br>- Blood pressure systolic ≥130 mmHg or diastolic ≥85 mmHg or antihypertensive therapy or previously diagnosed hypertension<br>- Fasting blood glucose ≥5.6 mmol/L (≥100 mg/dL) or glucose-lowering therapy or previously diagnosed type 2 diabetes mellitus

A1C, glycated hemoglobin; NGSP, National Glycohemoglobin Standardization Program; DCCT, Diabetes Control and Complications Trial; FPG, fasting plasma glucose; OGTT, oral glucose tolerance test; T2DM, type 2 diabetes mellitus; LDL-C, low-density lipoprotein cholesterol; HDL-C, high-density lipoprotein cholesterol; TG, triglycerides
