# Supplementary material for: Long-Term Metabolic Remission and Predictive Factors After Sleeve Gastrectomy and Roux-en-Y Gastric Bypass in an Asian Population
Source: J Clin Med. 2026 Feb 15;15(4):1539. doi: 10.3390/jcm15041539 (PMC12942549; doi:10.3390/jcm15041539)
Supplement: Supplementary file 1 [file jcm-15-01539-s001.zip › Table S2_181268.pdf]

Table S2. Definitions of the remission criteria for each comorbidity

Disease	Resolution criteria	Reference
Hypertension	- Improvement: Decrease in dosage or number of antihypertensive medications or decrease in systolic or diastolic blood pressure on the same medication (better control).<br>- Partial remission: Prehypertension blood pressure values (120–139/ 80–89) without medication.<br>- Complete remission: Normotensive (blood pressure <120/80) without antihypertensive medication. Medications used for another indication, such as beta-blockers for atrial fibrillation, should be documented but do not qualify as complete remission because of the dual therapeutic effect of some medications.	Standardized outcomes reporting in metabolic and bariatric surgery<br>(Surg Obes Relat Dis. 2015;11(3):489-506.)
T2DM	- Improvement: Decrease in dosage or number of antidiabetic medications or decrease in HbA1c on the same therapy (not mentioned in the consensus).<br>- Complete remission: Normoglycemia is restored (HbA1c <5.7%, FPG <100 mg/dL) without antidiabetic medications for at least 3 months.<br>- Partial remission: Glycemic indices in the pre-diabetic range (HbA1c 5.7–6.4%, FPG 100–125 mg/dL)	ADA, Endocrine Society, EASD, and Diabetes UK joint consensus statement on the definition of T2DM remission<br>(Int J Environ Res Public Health. 2019;16(17).)
Dyslipidemia	- Improvement: Decrease in the number or dose of lipid-lowering agents with equivalent control of dyslipidemia or improved lipid levels on the same medication regimen.<br>- Remission: Normal lipid profile without medication	Standardized outcomes reporting in metabolic and bariatric surgery<br>(Surg Obes Relat Dis. 2015;11(3):489-506.)
Metabolic syndrome	- Normalization of waist circumference or abnormal waist circumference plus one additional IDF criterion.	Remission of MS for the International Diabetes Federation [IDF] criteria<br>(Surgery. 2015;159.)

HbA1C, glycated hemoglobin; FPG, fasting plasma glucose
