# Supplementary material for: Long-Term Metabolic Remission and Predictive Factors After Sleeve Gastrectomy and Roux-en-Y Gastric Bypass in an Asian Population
Source: J Clin Med. 2026 Feb 15;15(4):1539. doi: 10.3390/jcm15041539 (PMC12942549; doi:10.3390/jcm15041539)
Supplement: Supplementary file 1 [file jcm-15-01539-s001.zip › Table S3_181268.pdf]

Table S3. Probability of remission outcomes by comorbidity and procedure

Comorbidities	Time (months)	Sleeve gastrectomy	Roux-en-Y gastric bypass
Type 2 diabetes mellitus	12	0.61 (95% CI: 0.43–0.73)	0.54 (95% CI: 0.37–0.66)
	24	0.64 (95% CI: 0.46–0.76)	0.54 (95% CI: 0.37–0.66)
	36	0.64 (95% CI: 0.46–0.76)	0.62 (95% CI: 0.43–0.74)
	48	–	0.66 (95% CI: 0.46–0.79)
Hypertension	12	0.45 (95% CI: 0.26–0.60)	0.33 (95% CI: 0.18–0.45)
	24	0.60 (95% CI: 0.36–0.75)	0.36 (95% CI: 0.20–0.48)
	36	0.60 (95% CI: 0.36–0.75)	0.40 (95% CI: 0.22–0.54)
	48	0.60 (95% CI: 0.36–0.75)	0.40 (95% CI: 0.22–0.54)
	60	0.60 (95% CI: 0.36–0.75)	–
Dyslipidemia	12	0.10 (95% CI: 0.00–0.19)	0.24 (95% CI: 0.10–0.35)
	24	0.14 (95% CI: 0.01–0.25)	0.26 (95% CI: 0.12–0.39)
	36	0.18 (95% CI: 0.03–0.31)	0.37 (95% CI: 0.19–0.51)
	48	0.18 (95% CI: 0.03–0.31)	0.41 (95% CI: 0.22–0.56)
	60	0.18 (95% CI: 0.03–0.31)	0.41 (95% CI: 0.22–0.56)
Metabolic syndrome	12	0.75 (95% CI: 0.64–0.82)	0.74 (95% CI: 0.64–0.81)
	24	0.84 (95% CI: 0.72–0.91)	0.85 (95% CI: 0.76–0.91)
	36	0.84 (95% CI: 0.72–0.91)	0.89 (95% CI: 0.80–0.94)
	48	0.94 (95% CI: 0.70–0.99)	0.89 (95% CI: 0.80–0.94)
	60	–	0.89 (95% CI: 0.80–0.94)

CI, confidence interval
