# Supplementary material for: Long-Term Metabolic Remission and Predictive Factors After Sleeve Gastrectomy and Roux-en-Y Gastric Bypass in an Asian Population
Source: J Clin Med. 2026 Feb 15;15(4):1539. doi: 10.3390/jcm15041539 (PMC12942549; doi:10.3390/jcm15041539)
Supplement: Supplementary file 1 [file jcm-15-01539-s001.zip › Table S4_181268.pdf]

Table S4. Comparison of lipid profile variables before and after bariatric surgery

	Total, n=173 (100%)			SG, n=128 (74%)			RYGB, n=45 (26%)		
	**Preop**	**Postop**	**p-value**	**Preop**	**Postop**	**p-value**	**Preop**	**Postop**	**p-value**
TG	132.50 (98.5, 183.0)	104.00 (70.0, 151.0)	<0.001	130 (105, 170)	102 (76, 128)	<0.001	154 (121, 211)	108 (79, 126)	<0.001
HDL	43.3 (36.4, 51.4)	50.4 (41.0, 59.3)	<0.001	43 (37, 54)	52 (43, 65)	<0.001	42 (38, 50)	46 (38, 51)	0.23
LDL	129.15 (99.3, 175.9)	127.55 (91.6, 177.4)	0.59	132 (95, 164)	145 (111, 180)	0.005	123 (108, 167)	102 (78, 115)	0.002
CHOL	184.5 (157.0, 215.8)	191.0 (156.0, 223.8)	0.28	190 (154, 219)	206 (164, 241)	0.001	181 (162, 217)	157 (140, 181)	0.001

Data are presented as median (IQR1, IQR3) unless otherwise indicated.

Variables include triglycerides (TG), high-density lipoprotein cholesterol (HDL), low-density lipoprotein cholesterol (LDL), total cholesterol (CHOL), preoperative (Preop), postoperative (Postop). Laparoscopic sleeve gastrectomy (LSG), laparoscopic Roux-en-Y gastric bypass (LRYGB)

All values are presented in mg/dL.
