# Supplementary material for: Long-Term Metabolic Remission and Predictive Factors After Sleeve Gastrectomy and Roux-en-Y Gastric Bypass in an Asian Population
Source: J Clin Med. 2026 Feb 15;15(4):1539. doi: 10.3390/jcm15041539 (PMC12942549; doi:10.3390/jcm15041539)
Supplement: Supplementary file 1 [file jcm-15-01539-s001.zip › Table S5_181268.pdf]

Table S5. Comparison of characteristics between patients who were lost to follow-up and those who continued follow-up at 5 years after bariatric surgery (n=238)

Characteristic	Lost to follow-up (n=127)	Followed-up (n=111)	p-value
Age (years), median (IQR)	29.6 (23.3, 39.8)	35.4 (30, 44.1)	<0.001
<30	65 (70.7)	27 (29.3)	<0.001
30–39	33 (41.2)	47 (58.8)	
40–49	20 (51.3)	19 (48.7)	
50–59	6 (30.0)	14 (70.0)	
≥60	3 (42.9)	4 (57.1)	
Female sex, n (%)	82 (52.2)	75 (47.8)	0.726
BMI (kg/m^2)			
<40	21 (44.7)	26 (55.3)	0.308
40–50	59 (53.2)	52 (46.8)	
>50	47 (58.8)	33 (41.2)	
Comorbidity, n (%):			
Type 2 diabetes mellitus	40 (47.1)	45 (52.9)	0.188
Insulin user	8 (57.1)	6 (42.9)	0.247
Duration of type 2 diabetes mellitus (months), median (IQR)	11.3 (1.9, 36.3)	13.7 (3.3, 51.0)	0.345
Hypertension	49 (46.7)	56 (53.3)	0.088
Dyslipidemia	81 (49.7)	82 (50.3)	0.125
Metabolic syndrome	97 (51.1)	93 (48.9)	0.208
Fatty liver, n (%)	115 (52.8)	103 (47.2)	0.698
Obstructive sleep apnea, n (%)			0.806
Mild	20 (46.5)	23 (53.5)	
Moderate	22 (56.4)	17 (43.6)	
Severe	67 (53.6)	58 (46.4)	
Primary snoring	16 (55.2)	13 (44.8)	
Gastroesophageal reflux disease, n (%)	11 (50.0)	11 (50.0)	0.914
Operation			0.035
Sleeve gastrectomy	95 (58.3)	68 (41.7)	
Roux-en-Y gastric bypass	32 (42.7)	43 (57.3)	

Data are presented as n (%) unless otherwise indicated.

CI, confidence interval; SD, standard deviation; IQR, interquartile range; BMI, body mass index.
